# Accommodation, slip inversion, and fault segmentation in a province-scale shear zone from high-resolution, densely spaced wide-aperture seismic profiling, Centennial Valley, MT, USA

**DOI:** 10.1038/s41598-019-45497-1

**Published:** 2019-06-25

**Authors:** Pier Paolo G. Bruno, Claudio Berti, Frank J. Pazzaglia

**Affiliations:** 1 Khalifa University, Department of Earth Sciences, P.O. Box 127788, Abu Dhabi, UAE; 2 Lehigh University, Department of Earth and Environmental Science, 18015 Bethlehem, PA USA; 3 Idaho Geological Survey, Moscow, ID 83844 USA

**Keywords:** Geophysics, Structural geology, Tectonics, Seismology

## Abstract

We acquired a ~9-km long, high-resolution reflection seismic profile in the Centennial Valley, Montana, to better understand the kinematics of basin bounding faults and their role in accommodating proposed right-lateral shear in the Northern Basin and Range adjacent to the Yellowstone hotspot. In pursuing these goals, our findings have also shed light on the development of hanging wall stratigraphy and seismic hazards for this part of the SW Montana seismic belt. Here we present the profile and a working interpretation that identifies fault inversion, and an oblique, anticlinal accommodation zone linking the Centennial and Lima Reservoir faults in the Centennial Valley. These interpretations are consistent with seismicity and GPS-geodetically observed right-lateral shear aligned with the Centennial Valley north of the Yellowstone hotspot. Data were acquired using dense, wide-aperture arrays and illuminate the subsurface stratigraphy and faults down to ~1200 m, showing that the basin is a half-graben with a southern depocenter driven by the listric geometry of the north-dipping Centennial fault. Reflectors onlap basement highs with growth geometry against these faults. Our interpretation of a bright basal reflection as the Timber Hill Basalt (~6 Ma) or related flow, is consistent with a late Miocene – Pliocene inception of the basin proposed by other research. We also note a small inversion structure that we interpret as local evidence of transpression in the shear zone. This transpression is part of the accommodation zone and seismogenic faults including the Lima Reservoir fault that has well-expressed Holocene surface ruptures a few kilometres west of the seismic line along the northern edge of the Centennial basin.

## Introduction

**Figure 1:**
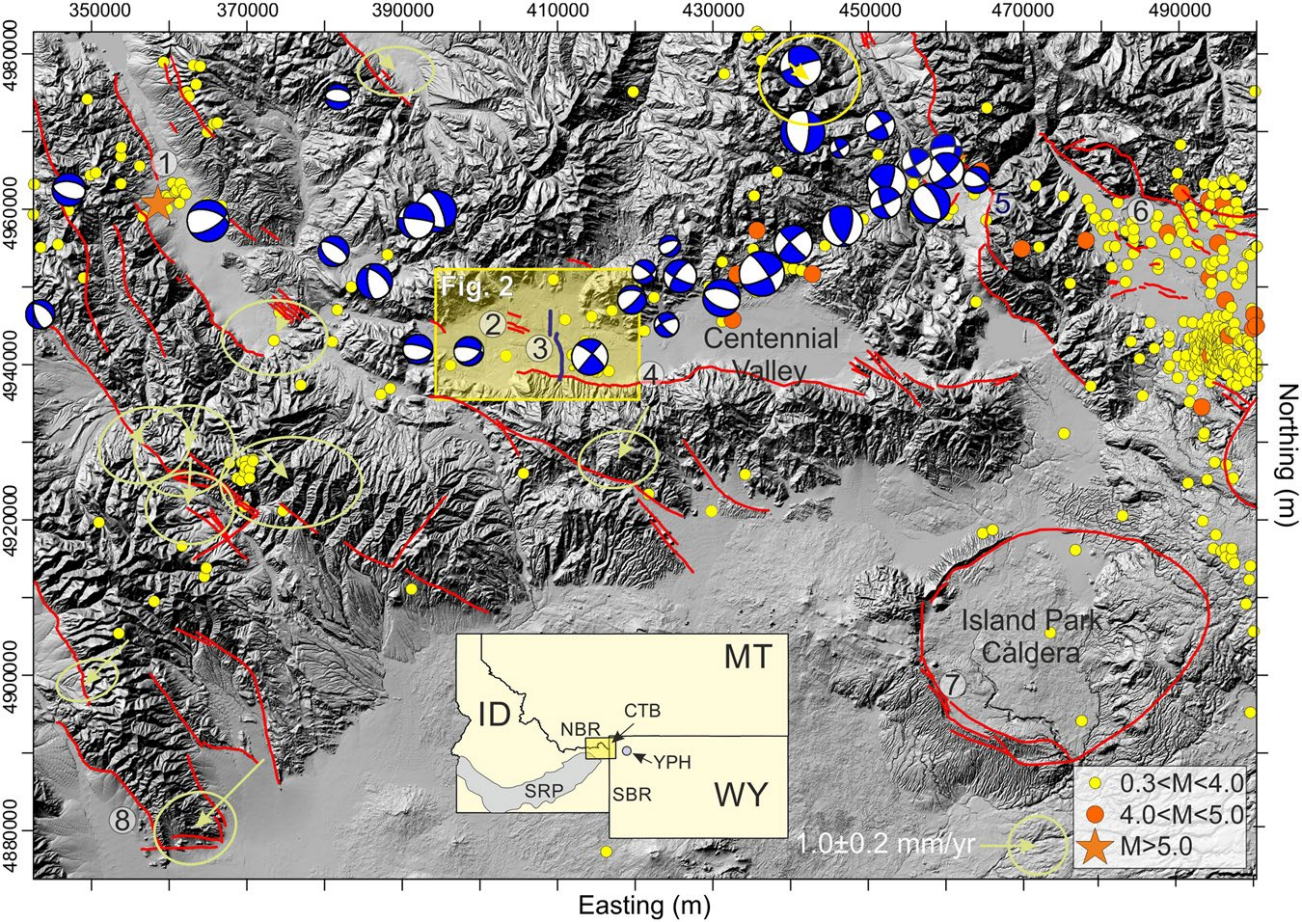
Shaded relief map of the Centennial Tectonic Belt (CTB) in the vicinity of the Centennial Valley showing major Quaternary faults^66,67^ in red, earthquakes and focal mechanisms^5^ as circles and stars (see legend), GPS velocity vectors with error ellipses^5^ in olive, and our seismic lines as the bold blue lines in the yellow shaded box that indicates the outline of Fig. 2. Symbol explanation: (1) Red Rock fault; (2) Lima Reservoir fault; (3) Seismic lines; (4) Centennial fault; (5) Madison fault; (6) Red Canyon fault; (7) Island Park Caldera ring faults; (8) Beaverhead fault. Quaternary fault locations^66^ and earthquake occurrence^67^ from 1973 to 2015 (yellow circles 0.3 < M < 4; red circles: 4 < M < 5; red star: M > 5). Horizontal Velocities of GPS Data From 1998 to 2014 in a Stable North American Reference Frame^5^. Inset shows the relative position of this figure with respect to the Yellowstone hotspot (YPH) and the Snake River plain (SRP). Topography from the U.S. Geological Survey (USGS) National Elevation Data set^68^ at 30 m resolution; coordinate system: North American Datum [NAD] 27, Universal Transverse Mercator [UTM] zone 12N).

More than a decade of continuous and campaign GPS geodesy of the greater Yellowstone region (Fig. 1) has generated a surface velocity field used to test geodynamic models of Northern Basin and Range (NBR) extension. These models include a 40–45 km-wide, province-scale, right-lateral shear zone called the Centennial shear zone (CSZ) that separates the NBR, Snake River Plain (SRP), and Southern Basin and Range (SBR) in the wake of the Yellowstone hotspot^1–5^. Seismicity across the CSZ reveals distributed deformation due to strike-slip faulting, distributed simple shear, regional-scale rotation, or some combination thereof. A significant implication of the geodetic and earthquake seismology work is that opposing-polarity normal faults mapped as the basin-bounding structures of the Centennial Valley, southwest Montana, have linked up at depth to function as a major right-lateral shear zone separating eastward movement of the Northern Rockies from westward movement of the SRP and NBR with crustal strain rates ranging from ~1–3 mm/yr^5^. These GPS-geodetic rates are an order of magnitude faster than the rate of fault slip obtained by paleoseismic data on faults in the Centennial tectonic belt (CTB^6–13^). The globally-recognized discrepancy of GPS, geologic, and co-seismic slip rates across major shear zones is an ongoing challenge in defining seismic hazards^14^.

**Figure 2:**
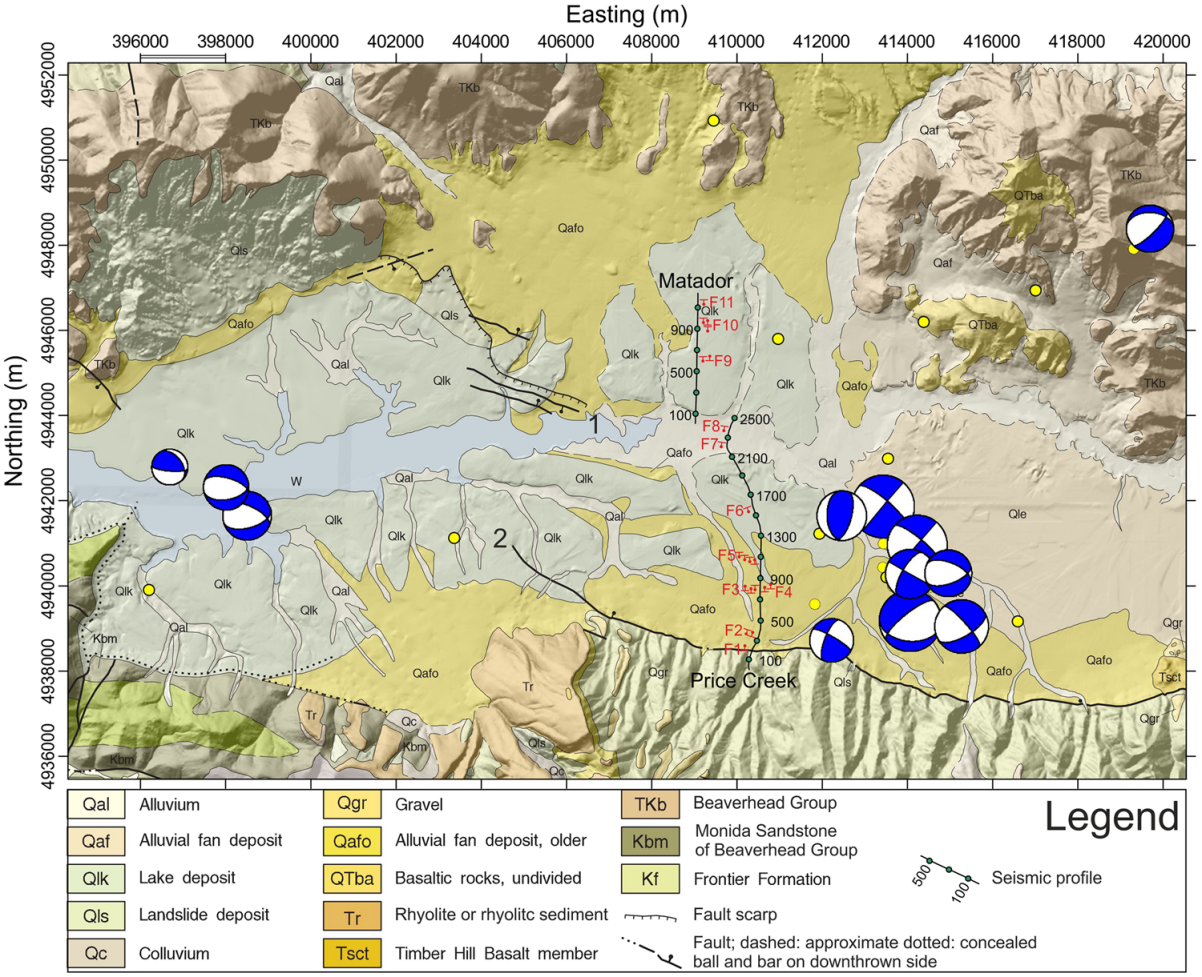
Geologic Map of the western sector of Centennial Valley (modified from Lonn *et al*.^51^), draped on shaded relief topography, and showing our two seismic lines, named the Matador line for the northern segment and the Price Creek line for the southern segment, major mapped faults^66,67^, recent earthquakes^5^, (yellow circle 0.3 < M < 4), and fault-plane solutions for selected earthquakes^5^. Symbols: (1) Lima Reservoir fault; (2) Centennial fault. Green dots plotted along the seismic lines are CDP locations annotated every 400 m. CDP numbers increase from south to north. The approximate position of vertical projections of the interpreted faults are shown with red symbols along the seismic profiles. The dot indicates the dip direction of the fault plane. Shaded relief map at 30 m resolution made using DEM data from the USGS National Elevation Data set^68^; coordinate system: North American Datum [NAD] 27, Universal Transverse Mercator [UTM] zone 12N).

High-resolution seismic reflection profiling, coupled with geomorphology and structural geology can be used to estimate the coseismic behaviour and kinematic evolution of active faults and define their otherwise inaccessible structural and kinematic evolution at millennial timescales. A recent example of this kind of work in the SBR province is provided by Bruno *et al*.^15^, who were able to image a ~3-km wide, off-fault damage zone characterized by distributed deformation along small displacement faults across the surface rupture of the 1934 Ms 6.6 Hansel Valley earthquake (Utah, USA). Building on that success, here we present results of a high-resolution, north-south-oriented seismic profile acquired, with the same field technique and instrument used by Bruno *et al*.^15^, across the Centennial Valley west of Yellowstone at a location capable of imaging an accommodation zone associated within the CSZ (Figs 1 and 2). Our surveys provide the first, high-resolution seismic image of the Centennial Valley depicting a ~1200 m deep, asymmetrical graben filled by high-amplitude, high-frequency, and high-continuity reflections above an overall low-reflectivity basement showing features consistent with syn-tectonic deformation and erosion. Furthermore, the data show the geometry of the Centennial Fault and the Lima Reservoir Fault as they enter the accommodation zone, and define a velocity structure for sediments within the basin. We use these results to evaluate the geometry and kinematics for a part of the CSZ, inform general models for fault growth, interaction, and linkage in an evolving accommodation zone^16–22^, and contribute to our understanding of the seismic hazards in the CTB, particularly given the discrepancy of paleoseismic and geodetic rates of crustal strain^14,16,17^.

## Geological Setting

The Centennial Valley is located in southwest Montana north of the SRP and on the NW flank of the Yellowstone hotspot (Fig. 1). The floor of the valley lies at an elevation of ~2000 m and is bound to the south by the 3000-m-high Centennial Mountains that form the continental divide. The valley is characterized by shallow lakes and marshes in its eastern extent that currently drain west into the Lima Reservoir, then the northwest-flowing Red Rock River, a tributary to the Beaverhead River and ultimately the Missouri River. Lima Reservoir is dammed at a narrow point in the course of the Red Rock River. Past landslides may have naturally blocked or restricted river flow near the location of the reservoir, which when combined with different climatic and/or basin subsidence conditions, caused the Centennial Valley to fill with a lake exceeding the size of the reservoir at various periods of time through the Pliocene and Pleistocene^23^.

Centennial Valley is part of the NBR which formed following a long history of Cenozoic extension that dismembered the thick crust built during Laramide and Sevier deformation in the Northern Rockies^1,10,24–29^. Coincident in part with Eocene shortening, parts of southwestern Montana also began to extend along mid-crustal detachments, forming core complexes. As extension continued, high-angle faults that sole into the detachment formed, creating elongate, NE-SW-oriented basins. These late Eocene and Oligocene basins filled with a thick pile of Challis volcanic and volcaniclastic rocks. The arrival of the Yellowstone hotspot, the largest geophysical anomaly in North America whose initiation and evolution is deeply related to the NBR^1,30–32^, at ~16 Ma initiated a new phase of extension. This younger phase inverted some of the older basins and established the current NW-SE tectonic grain that terminates to the north against the Snowcrest-Blacktail Laramide block. Yellowstone also initiated a new, distinctive suite of bimodal volcanism, producing flows that may show up as key seismic reflections in the Centennial seismic line.

Major earthquakes such as the 1983 (Mo 6.9) Borah Peak, Idaho earthquake, which ruptured the central segment of the Lost River fault with oblique normal slip, including a 17% sinistral component^33^, indicates that NBR extension continues. This most recent phase of stretching is characterized by a right-lateral shear couple with localized transtension and transpression focused on the transition from the NBR to the SRP. Where the NBR meets the SRP, gravity and seismic-reflection data suggest that significant normal-fault offsets do not extend further south^34,35^. The decrease in peak elevation of mountain ranges within the CTB toward the eastern SRP is interpreted to reflect flexural downwarping due to subsidence of the eastern SRP^1,36–38^. The Centennial Valley is favourably oriented to accommodate the implied shear across its southern boundary with the SRP.

The south-dipping Lima Reservoir fault and north-dipping Centennial fault bound the Centennial Valley to the north and south respectively (Fig. 2). The distribution of Centennial fault scarps and surface ruptures suggests that it is composed of a series of NW-trending, left-stepping, en-echelon segments compatible with distributed dextral shear, which gives the appearance of an overall east-west trend of a broader fault zone^26,39,40^. The eastern end of the Lima Reservoir fault nearly reaches the same longitude as the western end of the Centennial Fault, forming two oppositely dipping normal faults interpreted to accommodate right-lateral transtension^12,41–43^.

All of these active faults are part of the CTB where earthquakes with normal fault-plane solutions are mostly concentrated on NW-trending faults^44–46^. Near the Lima Reservoir and Centennial faults and within the Centennial shear zone, fault-plane solutions are mixed, with a variety of oblique normal slip, strike slip, and even oblique reverse slip^47,48^ (Figs 1 and 2). Evaluation of focal depths and N-dipping nodal planes suggest that only eight events (M < 4.4) may be associated with slip on the Centennial fault^48^. The majority of the fault plane solutions (M < 4.6) are strike slip and have right-lateral motion along NE-trending nodal planes. They are located within a NE-trending zone of seismicity that is oblique to the E trend of the Centennial fault. The fastest lateral shearing is closest to the Yellowstone Plateau, where fault-plane solutions with components of right-lateral strike slip are documented within a NE-trending zone of seismicity.

Rocks and sediments exposed in the footwall of the Centennial fault^39,40,49,51^ (see also Supplementary Fig. S1) provide a general stratigraphic framework to interpret seismic reflection data in the hanging wall block that has neither exposure, nor publicly available well data. The Centennial footwall is cored by Precambrian basement rocks unconformably overlain by a passive-margin sequence of Paleozoic marine siliciclastics and carbonates. The subsequent, unconformable Mesozoic sequence is a mix of marine and continental siliciclastics and carbonates deposited in the Sevier foreland. These Mesozoic rocks are thickest in the western Centennial Mountains where the Cretaceous Frontier Formation composes the bedrock. Across the valley to the north and at the foot of the Snowcrest Range, basal conglomerates of the late Cretaceous-early Tertiary Beaverhead Group unconformably cover Paleozoic and older Mesozoic rocks^50^. Folds in the Beaverhead Group are evidence for syn-deformation deposition during the Sevier orogeny^43^. Unconformably overlying the Frontier Formation and Beaverhead Group are mid-late Cenozoic volcanic and volcaniclastic rocks of rhyolitic, andesitic, and basaltic composition with Challis and later Yellowstone-SRP affinities. Of particular note are Late Tertiary rhyolite, rhyolitic volcaniclastics, and basalt, the latter of which may be generally correlative to the Timber Hill basalt (6 Ma^51^; T_SC_ in Supplementary Fig. S1), that are draped across the Centennial Fault footwall at the western terminus of the fault, only a few kilometres both east and west of our seismic line (Fig. 2). The younger part of this section is dominated by Quaternary alluvial, alluvial fan, landslide, and lake deposits that are exposed at the basin margin and projected to underlie the Centennial Fault hanging wall.

## Data and Methods

Our investigation consists of two N-S trending, partially overlapping profiles (Fig. 2). The assembled profile takes advantage of the only north-south oriented dirt road (Price Creek Road, not shown on Fig. 2) that traverses the alluvial fans in the hanging wall near its southern end to obliquely intersect the Centennial Fault. The southern profile (the Price Creek line) originates ~400 m south of the surface expression of Centennial Fault, in the footwall block, and covers ~half of the valley. The northern profile (the Matador line, Fig. 2) is offset ~900 m the west to account for the bridge crossing of the Red Rock River. It begins on the north side of the river in an open field of the Matador Ranch, and then continues to follow a dirt road oriented due north (North Valley Road, also not shown on Fig. 2). The latitude coordinates of the two profiles overlap for about 300 m. In total, the assembled profile stretches for about 9,300 m, covering more than ¾ of the valley width. To ensure very dense spatial sampling, the lines were collected with 5-m spacing of geophones and sources.

For the P-wave seismic source we used a 6400 kg high-resolution vibrator (IVI-MiniVib t7200^®^) that was progressively moved at 5 m intervals within the 168-channel geophone array made of 4.5-Hz vertical geophones spaced at 5 m. At each vibration point, we stacked two 15-s-long linear sweeps starting at 5 Hz and ending at 200 Hz. Tight spacing of both geophone and vibration points ensured a very regular and dense subsurface coverage, with common midpoints (CMP) spaced at 2.5 m (see Supplementary Fig. S2).

A thorough description of the processing phase is beyond the scope of this paper; therefore, we limit the description to the major steps, details of which can be found in Yilmaz^52^ and references therein. The workflow used for seismic data processing is detailed in Supplementary Fig. S3. Supplementary Table S3 further details the seismic data processing stream and parameters used.

Four representative common-shot panels (Supplementary Fig. S2) show excellent data quality in the valley, with high-frequency reflections (i.e. dominant frequencies of ~80–120 Hz) down to ~1 s TWT. Moreover, clear first arrivals characterize the entire offset range. Signal-to noise ratio degrades across the Centennial fault, because of static problems caused by the rugged topography and by laterally heterogeneous near-surface velocities (Supplementary Figs S2 and S4). In fault-bounded basins, surface-consistent refraction statics can play an important role for improving the quality of the stack^15^. For this purpose, first arrival travel-times were picked on about ~97,200 waveforms and checked for consistency using the reciprocity rules of Ackermann *et al*.^53^. We used first-arrivals also to estimate a detailed, near-surface P-wave velocity model using the turning-ray tomography technique^54^ (Supplementary Fig. S4). A preliminary velocity model was estimated using a time-delay refraction method and discretized on a 10 × 10 m grid size. The travel-time residuals between observed and theoretical travel times were iteratively inverted using a SIRT-based method^55^.

Spiking and predictive deconvolution allowed us to improve the temporal resolution within the seismic reflection data and to attenuate short-period multiples. Semblance-based velocity analysis was performed by picking the maxima of the semblance function computed across hyperbolic trajectories^56^ on selected CMP super-gathers. The velocity models and the CMP stacks were refined by multiple cycles of residual statics corrections^57^ followed by an additional velocity analysis phase. To reduce the error on reflector position and shape introduced by the common mid-point approximations the final stacks were depth migrated using the Kirchhoff algorithm^52^. In order to provide a smoothed P-wave velocity model for migration, the final stacking velocity model was converted in interval velocity vs. depth using the Dix formula^58^ and applying smoothing functions to the results, in order to avoid blocked interval velocity functions that can have large velocity discontinuities between blocks.

**Figure 3:**
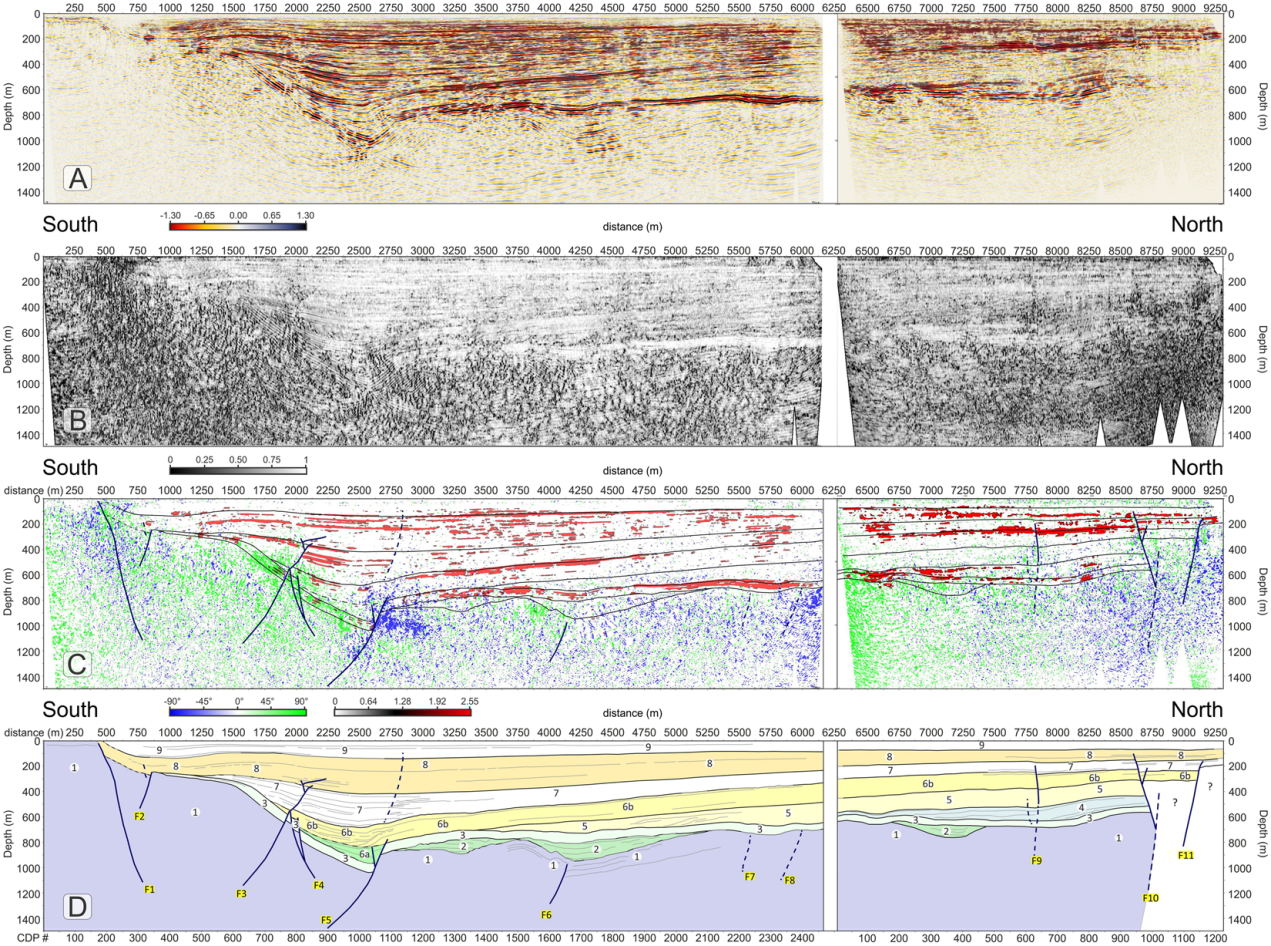
(**A**) seismic amplitudes of the Price Creek (left) and Matador (right) lines without vertical exaggeration plotted with a gradational red-white-blue palette with positive amplitudes shown in blue. (**B**) similarity attribute, plotted using a gradational grey palette; black colours highlight areas of the profile with the lowest similarity. (**C**) overlap of energy attribute and dip-angle attribute. Red colours highlight areas that have energy greater than 80% of maximum energy. Energy values < 80% and dip attributes less than ±30° are not plotted. Faults (in blue) are located by matching off-set seismic amplitude features with dip, energy and similarity attributes. The boundaries of the interpreted seismic units are also shown in black. (**D**) Stratigraphic interpretation scheme for the two profiles. Faults are coloured in blue. Dashed lines indicate faults with offsets below the resolution limit that were interpreted mainly based on seismic attributes. For unit descriptions see the legend of Fig. 2 and refer to the text for an explanation of the model.

Different seismic attributes were tested in a trial-and-error mode on the final migrated section and we selected and describe three that provided additional useful elements to constraint our seismic interpretation of the profiles^59^, i.e.: 1) similarity; 2) energy and 3) dip angle. The similarity attribute (Fig. 3B) is a multi-trace attribute particularly useful to detect areas with low lateral coherence such as fractures and faults. The energy attribute (Fig. 3C) is a measure of reflectivity strength within the chosen time-gate, therefore it is useful to locate bright spots and to delineate the unconformities, as often they are characterized by a strong impedance contrast. Finally, the dip-angle attribute (also plotted in Fig. 3C with a different colour palette and overlain with the energy attribute) provides the apparent angle of dip (in degrees) of seismic features within the profiles.

### Basin seismic stratigraphy and faults

**Figure 4:**
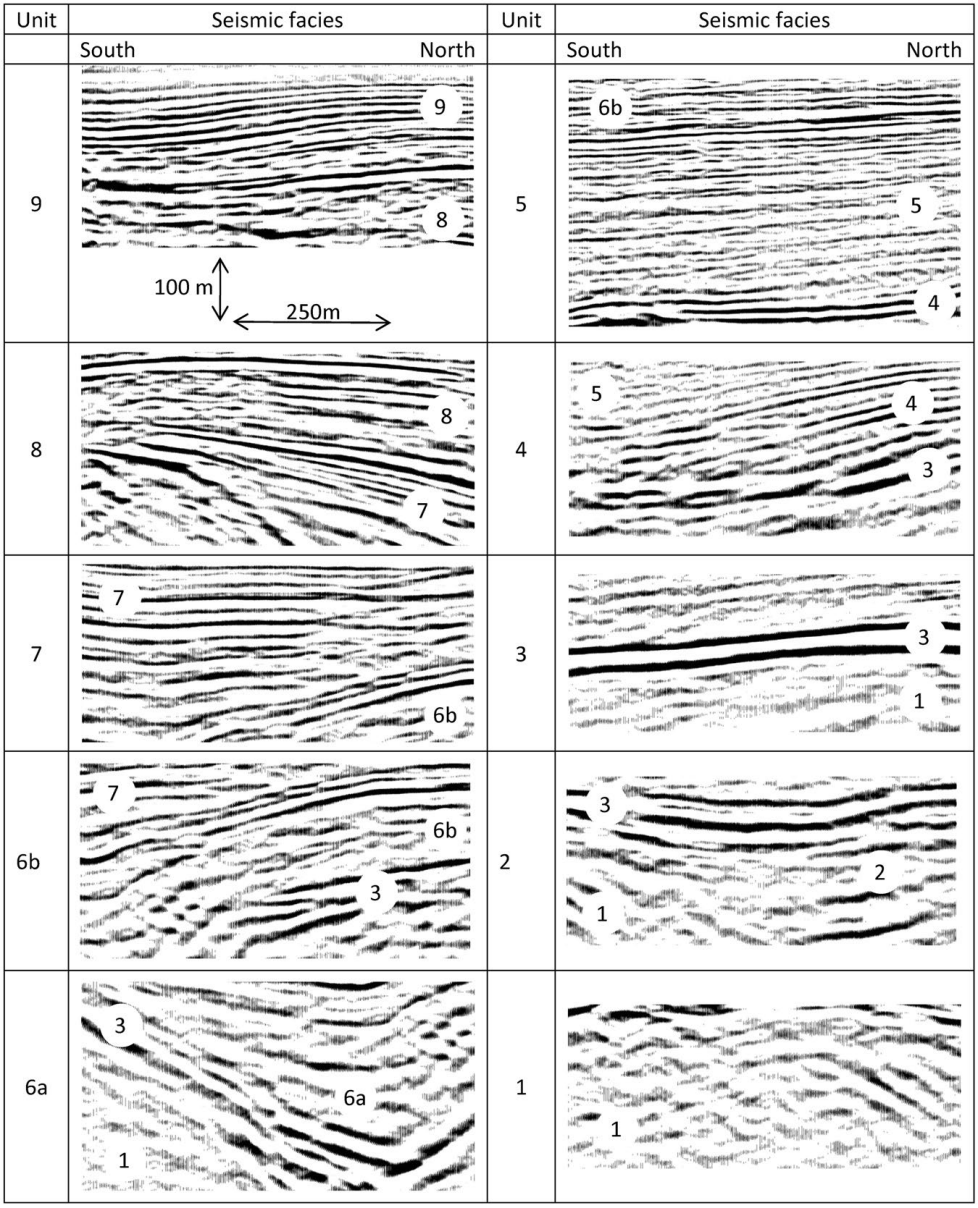
Main facies characteristics of the seismo-stratigraphic units.

The final migrated profiles of the Price Creek and Matador lines are shown in Fig. 3. The two profiles show similar seismic character when plotted side-by-side in Fig. 3A even though they are separated by ~900 m in the overlapping section. Our structural and stratigraphic interpretation of the post-stack depth-migrated seismic images, which is synthesized in the scheme of Fig. 4 is based on the comparison of the reflection character and configuration patterns (Table 1) from the seismic image in Fig. 3A and in Fig. 4 with the chosen seismic attributes (Fig. 3B,C) briefly explained above, and it is also guided by: (1) the results of the first-arrival seismic tomography, which provides complementary information near the surface (Fig. S2); (2) the surface geology^51^ and an exploration well drilled into the Centennial fault footwall^50^; and (3) geomorphic analyses of stream channels and Quaternary deposits^60^.

**Table 1:** Description of the reflective character and configurations of the seismostratigraphic units shown in Fig. 4.

Unit	Reflection character	Reflection configuration patterns & terminations
9	High-frequency, high amplitude, high-continuity.	Warped, roughly parallel, with divergent geometry to the south.
8	High to fair amplitude, high frequency, high to fair continuity.	Parallel to divergent, showing a mild angular unconformity (onlap) above unit 7.
7	Fair to high amplitude, fair frequency, fair continuity.	Subparallel to divergent with foreset geometry and onlap above unit 6b.
6b	Low amplitude, fair frequency, fair continuity.	Subparallel to prograding (sigmoid); downlaps above units 3 and 6a.
6a	Low- to fair-amplitude, low-frequency, dicontinuous.	Divergent, thickening towards the to north (interpreted as growth geometry).Onlap above the top of unit 3 (south).
5	Fair amplitude, high-frequency, high- to fair continuity.	Parallel to sub-parallel; downlaps above the top of unit 4.
4	Fair to high amplitude, fair to high frequency, fair continuity.	Diverergent to progradational; downlaps above the top of unit 3.
3	Couple of well distinct reflectors with high-amplitude and low-frequency.	Parallel configuration and unconformable contact (onlap – downlap, concordance) above units 2 and 1.
2	Low-ampitude,low-frequency, low to fair continuity.	Onlap above acoustic basement.
1	Low-amplitude, low-frequency, low-continuiy.	Acoustic basement. Erosional truncation at the top. Complex to subparallel internal reflective configurations - deformed.

### Structural analysis

The seismic amplitude plot in Fig. 3A shows excellent data quality within the valley achieving a penetration down to ∼1200 m that illuminates the overall shape of the basin. The clear difference between the basin fill and the basement is well portrayed by the similarity plot of Fig. 3B, where the recent basin fill overall is characterized by predominance of white, meaning higher values of similarity, with respect to the valley basement. The similarity plot also shows some areas within the basin that have a different behaviour such as low-similarity patches coincident with the surface expression of Centennial Fault (at a metric distance of 200–700 m) and at metric distances 1330, 1500, 1750–2250, 2500, 4100, 4500, 5500, 7800, 8600 and 9200 m where they define an intricate network of anastomosed, sub-vertical black segments likely to be caused by faults and fractures.

We interpret faults (Fig. 3C) only when we can see clear and consistent reflection offsets in the amplitude plot (Fig. 3A) that are also associated with amplitude reductions/anomalies that are marked by a low-similarity (i.e. black) attribute (Fig. 3B). At the same time, we note that high-angle fault/fracture planes show high values of the dip attribute, and in particular they are visible in Fig. 3C as blue clusters if south-dipping or green clusters if north-dipping. Generally, these blue and green clusters also display a good spatial correlation with the low-similarity (i.e. black) patches in Fig. 3B. Based on this strict matching requirement and on consistent reflector offsets on amplitude plots as a discriminant factor, our threshold for fault detection was represented by the vertical resolution limit (i.e. the Rayleigh criterion, where the seismic “measure” is a ¼ wavelength, which in our case ranges between ∼3.3 m at the surface to ∼16 m at ∼100 m deep). Figure 3B,C show that all interpreted faults are generally located within anomalous patches of dip and similarity attributes consistent with a high probability of fractures and small offset above the fault tips.

**Figure 5:**
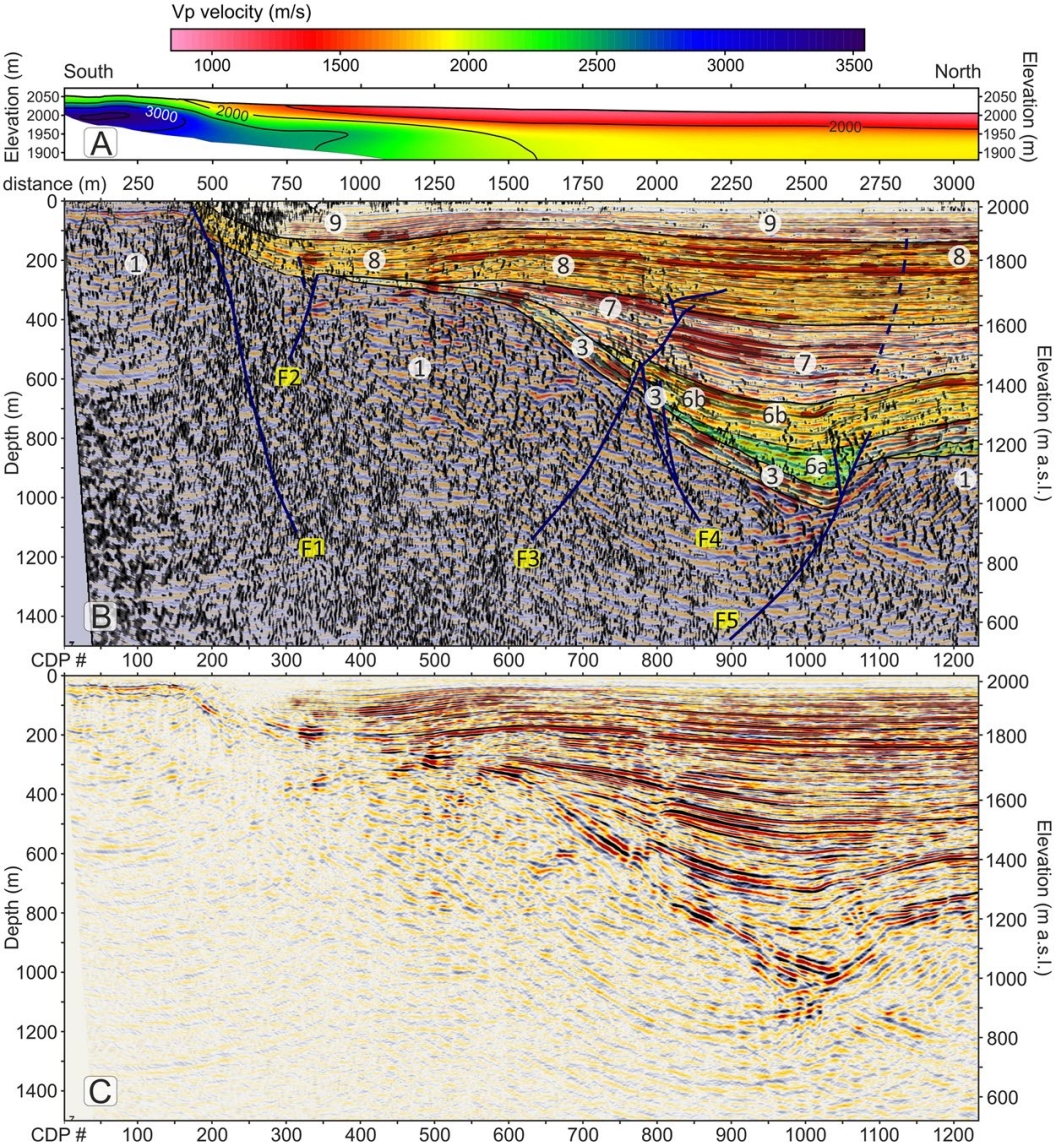
(**A**) Detail of the southern part of the Price Creek Vp refraction tomography profile (metric distance 0–3100 m), plotted without vertical exaggeration. (**B**) Detail of the southern part of the Price Creek seismic reflection profile (metric distance 0–3100 m), in which similarity and energy attributes are overlain with seismic amplitudes and plotted without vertical exaggeration. Seismic amplitudes are plotted with a gradational red-white-blue palette (see Fig. 3A); positive amplitudes are in blue. Similarity attributes are plotted using a gradational grey palette (see Fig. 3B); black colours highlight areas of the profile with the lowest similarity. Similarity attributes ≥0.2 are made transparent. Energy attribute is plotted with a gradational red-black-white palette. Energy values < 80% are made transparent. The structural & stratigraphic interpretation of Fig. 3D is also overlain with seismic attributes. (**C**) Detail of the uninterpreted southern part of the Price Creek line.

**Figure 6:**
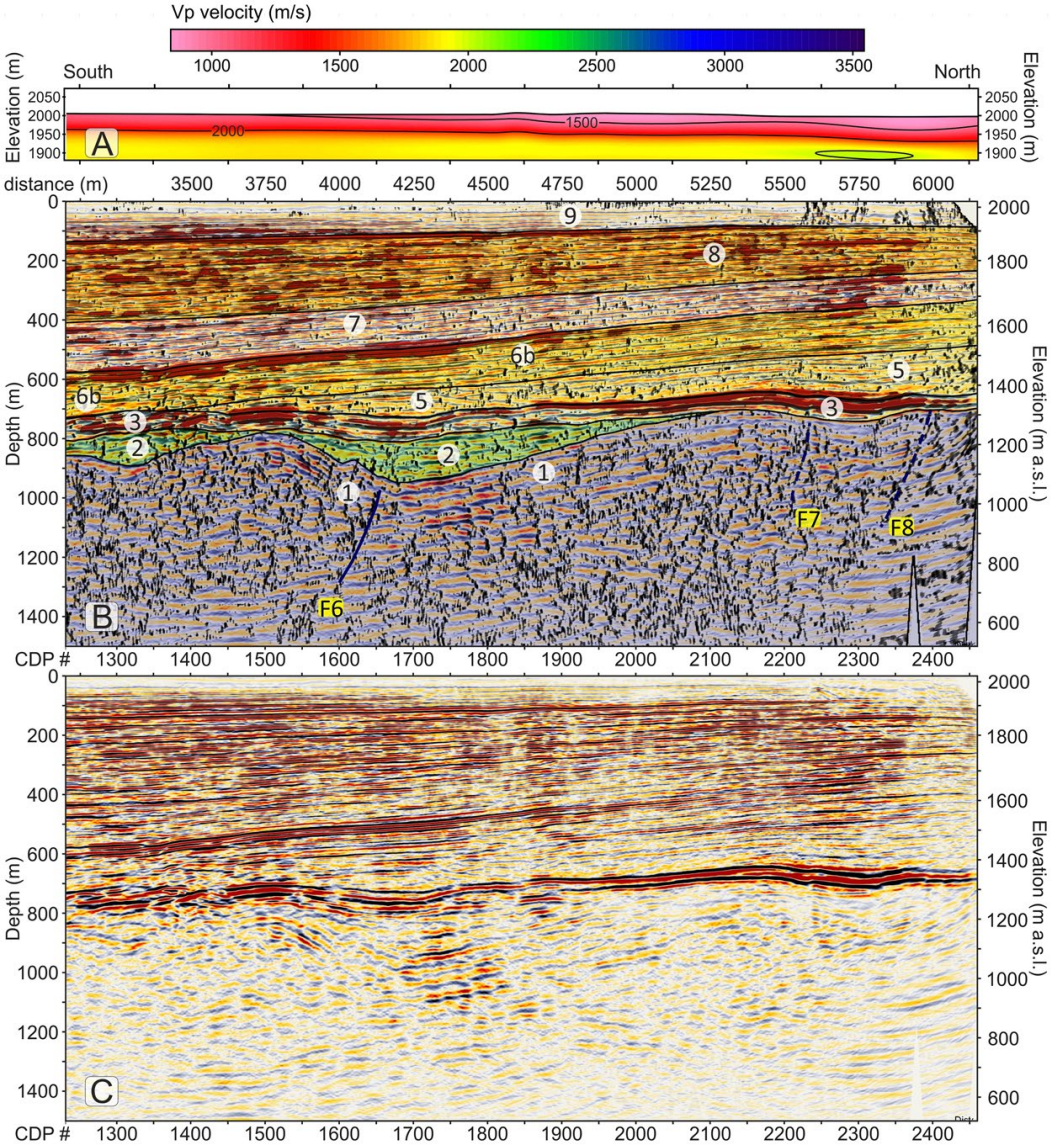
(**A**) Detail of the northern part of the Price Creek Vp refraction tomography profile (metric distance 3100–6150 m), plotted without vertical exaggeration. (**B**) Detail of the northern part of the Price Creek seismic reflection profile (metric distance 3100–6150 m), in which our interpretation, similarity and energy attributes are overlain with seismic amplitudes and plotted without vertical exaggeration. Plotting attributes are as in Fig. 5B. (**C**) Detail of the uninterpreted northern part of the Price Creek line.

**Figure 7:**
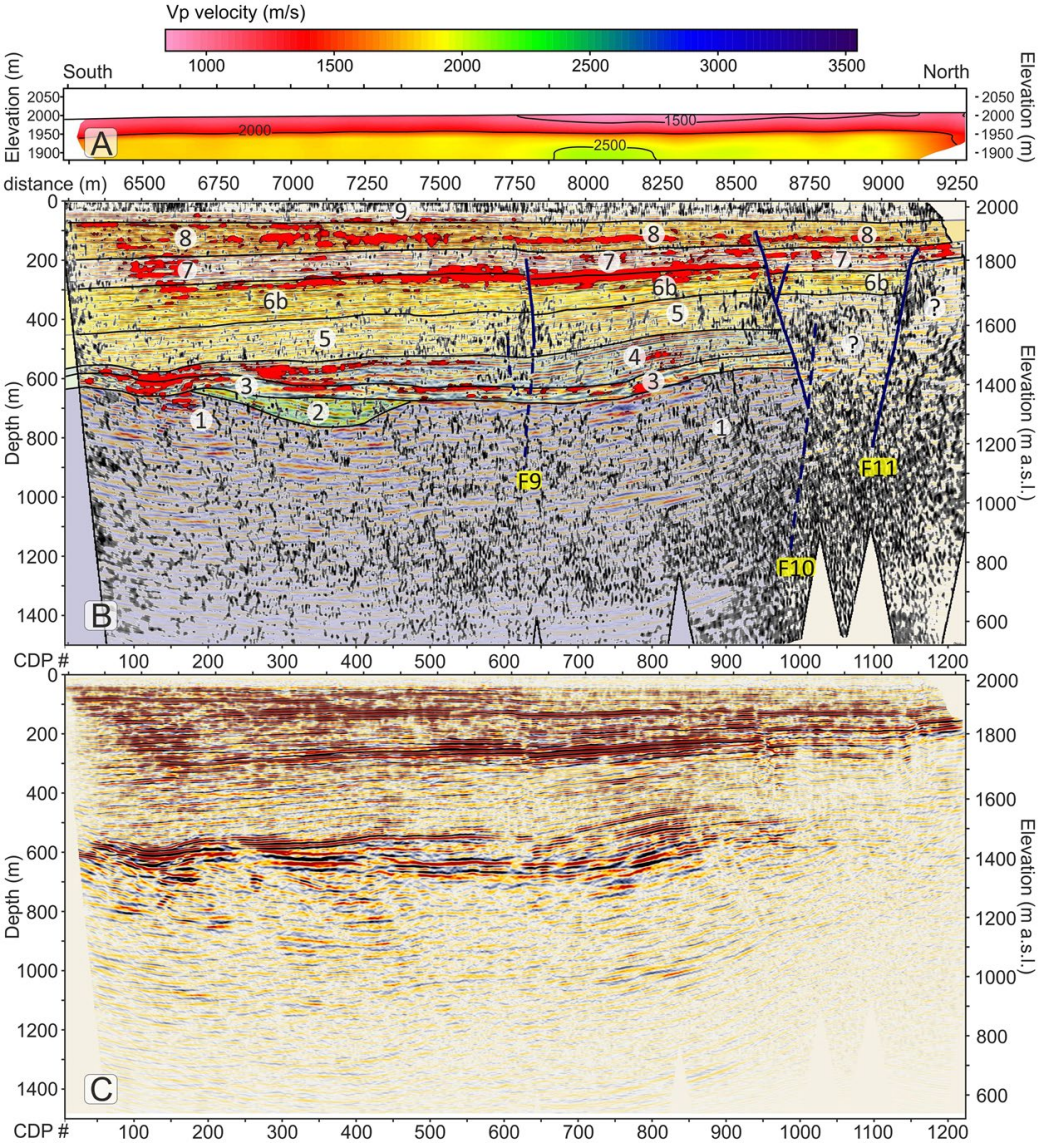
(**A**) Matador Vp refraction tomography profile, plotted without vertical exaggeration. (**B**) Matador seismic reflection profile, plotted without vertical exaggeration. Similarity; energy attributes and seismic interpretation are overlain with seismic amplitudes. Plotting attributes are as in Fig. 5B. (**C**) Detail of the uninterpreted Matador seismic line.

Overall, we were able to detect eleven significant faults across the two profiles (see Figs 5–7 for a higher detail). The first five of them (Fig. 5) form a complex set of conjugate splays that are genetically linked to the Centennial Fault, whereas faults F_10_ and F_11_ (Fig. 7) are interpreted to be the eastward extension of the Lima Reservoir Fault (Fig. 2). The valley depocenter in Fig. 5 is asymmetrically located at ∼2600 m from the southern end of the line, more than 3 km south from the actual location of Red Rock River. The depocenter position and shape is clearly linked to the activity of Centennial fault (F_1_) and its conjugate strands. However, from an initial inspection of Figs 3 and 5 the largest throw is not associated with the fault strand generating the surface expression of Centennial fault (F_1_) but with the conjugate sets labelled as F_3_-F_5_ and located ∼2000 m north of the surface rupture. From these figures, it is also clear that collectively, the Centennial fault zone controls the overall depositional architecture of the southern part of the basin. Major reflector packages show growth relationships that dip toward the depocenter and thin towards the northern end of the basin. Growth relationships are most evident by warped reflectors close to the fault planes and onlapping relationships with unconformities (Fig. 4 and Table 1). The southern margin of the basin (metric distance 1000–2000 m) shows a broad deflection of the of the most recent units (Fig. 5: U_8_-U_9_), associated with fault block rotation between the fault branches F_1_-F_5_. As discussed later, the warping of U_8_ and U_9_ can be associated with the reactivation of fault F_3_ in compression, as supported by the inverted offset of reflectors in Unit U_7_ and the presence of growth geometries in U_9_ between meters 1000 and 1500.

### Seismic stratigraphy

The overall heterogeneous character of the reflections is used to define packages of (1) high-to fair-continuity, high-frequency low- to fair-amplitude reflections separated by (2) very high-amplitude reflections (Fig. 4) that match the location of the high-energy (i.e. red) attribute plot of Fig. 3C. We use reflectivity strength and geometrical relationships as our key criteria in identifying stratigraphic unconformities that are known to be characterized by a strong impedance contrast. The unconformities defined using these criteria show high to fair lateral continuity within the basin and provide a basis for the delineation of major, unconformity-bound seismic stratigraphic packages identified using classical seismic sequence analysis schemes^61^ (Figs 3D and 4).

The definition of seismic stratigraphic packages is also guided by a line drawing of industry seismic data constrained by a well that penetrates the footwall of the Centennial Fault south of the terminus of our line^50^. Our correlation of lithostratigraphic units in Figs 2 and S1 to unconformity-bound seismic stratigraphic packages in Figs 5–7 is based on this line drawing and the regional geology. We define nine unconformity-bound seismic stratigraphic units based on the brightest reflections in the section highlighted by the energy attribute (Fig. 3C). Reflection configuration patterns within the seismic stratigraphic units show typical stratal geometries including onlap, offlap, concordance, and growth relationships with faults that further guide our genetic interpretations (Fig. 4).

The deepest, continuous bright reflection (Fig. 3A, 6 and 7) is interpreted as the unconformity at the top of the Palaeozoic and Mesozoic bedrock that was exposed and cut during Laramide/Sevier shortening and uplift. Unit 1 (Figs 5–7) is the deformed Palaeozoic and Mesozoic bedrock beneath the bright basal unconformity. Unit 2 locally overlies the basal unconformity, is deformed, and interpreted to be the syn-tectonic late Cretaceous – early Tertiary Beaverhead Conglomerate that was sourced from the Sevier highlands to the west. Unit 2 just occupies the structural lows atop the basal unconformity and presents piggy-back geometry.

Unit 3 and its basal unconformity collectively define the brightest reflector-sediment package in our section that we interpret to be late Tertiary volcanic or volcaniclastic rocks such as the ancestral Yellowstone-SRP rhyolite and basalt that is mapped draping and dipping north across the Centennial footwall just west of the seismic line (Fig. 2, units Tsct, Tr, Tba, QTba). Some of these volcanic rocks, namely the basalt flow, are thought to be time correlative to the Timber Hill basalt of the Six-Mile Creek Conglomerate (T_SC_ in Supplementary Fig. S1), dated to be ~6 Ma^51^. If this correlation is correct, our Unit 3 and its basal unconformity represent an excellent time-stratigraphic horizon from which to measure rates of deformation from the late Miocene to the Present. The formation of the current Centennial Valley occurred after the deposition of Unit 3 and if the unconformity at its base is in fact 6 Ma, the long-term average slip rate on the Centennial Fault would be ~216 m/my (0.216 mm/yr), to account for ~1300 m of stratigraphic separation of unit 3 (~900 m of basin subsidence and ~400 m of footwall uplift) over that time span.

The initial subsidence of Centennial Valley, following emplacement of Unit 3 is recorded by a set of two stacked alluvial fans, Unit 4 and 5, both thickening to the North (Figs 6 and 7). Based only on regional stratigraphy and Quaternary fan provenance, we suggest that the proximal facies of Units 4 and 5 are probably reworked Beaverhead Formation and Six Mile Creek Formation conglomerate, sourced in the Lima Reservoir Fault footwall, and deposited as paleo-alluvial fan facies. Further south in the basin, these fans may interfinger with axial stream fluvial facies.

Unit 6 records a major change in fault activity and subsidence in the southern part of the basin with activation of the Centennial Fault (F_1_) and an antithetic normal fault (F_5_) and sediment contributed from both uplifted footwalls as well as an axial system (Fig. 5). Unit 6 shows growth along the Centennial fault system with a sub-Unit 6a being trapped in a narrow basin of the F_5_ hanging wall and sub-Unit 6b showing both concordant, prograding and locally warped reflectors indicative of a complete fluvio-lacustrine depositional system with lake sediments in the deepest part of the basin (Fig. 4). Subsidence of the Centennial fault hanging wall continued during deposition of Unit 7, with more material being contributed by fans off of the Centennial fault footwall which appears to displace the depocenter further north. Unit 7 also shows syndepositional deformation associated with faults F_3_, F_4_, and F_5_, the former of which begins to show inverted slip. Units 6 and 7 probably represent a mixed provenance of reworked Beaverhead and Six Mile Creek formations, sediment sourced from the uplifted Cretaceous through late Tertiary rocks in the Centennial fault footwall, sediment delivered from the east via the axial stream system, and eolian material.

Units 6 and 7 are likely early to middle Pleistocene in age. If most of the slip on the Centennial Fault occurred syn- and post Unit 6, the slip rate calculated above (~216 m/my) is a minimum. An early Pleistocene age for Unit 6 suggests that the slip rate would triple to ~650 m/my (0.65 mm/yr).

Units 8 and 9 show mostly concordant, gently warped reflections that onlap the basin margins, consistent with a phase of axial stream alluvial and lake sediment filling. There is some evidence between 1000 m and 1500 m in Figs 5 and 6 of thickness changes in Unit 9 which is a possible indication of syn-depositional growth strata above fault F_3_. Otherwise, packages of weakly-reflective, nearly-uniform-thickness reflections sandwiched between bright reflections are particularly evident in the central part of Unit 8. If these are lake facies, they may represent cycles of lake expansion and contraction following pluvial glacial and dry interglacial cycles in the middle and late Pleistocene as well as Holocene.

### Inversion structure and accommodation zone

Some of the faults in the Price and Matador lines show features consistent with reactivation of pre-NBR faults, fault-slip inversion, and progressive development of an accommodation zone. Discontinuous reflectors in Unit 1, interpreted as syn-Sevier/Laramide compressional structures, terminate against south-dipping, presumably north-verging thrust faults with dips similar to the younger faults that cut up section into the overlying units. NBR extension likely reactivated these earlier compressional structures in the development of the Centennial basin. Similarly, seismic units are offset by the northern-most faults, F_9_, F_10_, and F_11_, (Fig. 7) that are on strike with the projection of the Lima Reservoir Fault (Fig. 2). Oblique-slip motion on small normal faults generated by the eastward propagation of the Lima Reservoir fault tip towards the Matador line may help explain the poor resolution and small stratigraphic offset astride these faults.

In contrast, clear stratigraphic offset and south-stepping fault slip is evident for younger seismic units on faults F_5_, F_3_, and F_1_ (Fig. 5). The current mountain front, defined by F_1_, is evidently a youthful feature, formed by the western propagation of the westernmost segment of the Centennial Fault (Fig. 2). Similarly, the synthetic F_4_ fault is directly on strike with the Centennial fault segment that forms the mountain front east of the Price Creek line. As a result, the southward stepping of normal fault development seems to be consistent with westward propagation of the normal fault segments. As the Centennial fault segments propagate westward and the Lima Reservoir fault tips propagate eastward, they begin to overlap, forming the accommodation zone with opposing fault dips.

## Discussion

The south and westward propagation of the Centennial fault, the inversion structure, and the eastward propagation of the Lima Reservoir fault are all consistent with the proposed right-lateral shear on CSZ^3,5^ and the progressive counter-clockwise rotation of NBR extension initially oriented NE-SW to its current NW-SE orientation following the passage of the Yellowstone hotspot^6^. The left-lateral component of oblique slip on the Centennial fault zone results in curved fault segments that are oriented WNW, with segment boundaries characterized by a step in the fault to the south^39^. This geometry is reminiscent of a Riedel-shear fault segmentation model proposed by Menges^62^ for the Sangre de Cristo Mountains of northern New Mexico. For the Centennial Valley and mountain front, the E-W right-lateral shear zone (CSZ) generates WNW right-lateral Riedel-shear fault segments with the segment breaks characterized by left-lateral NNW-oriented cross-faults. The overlapping and offset tips of the fault segments locally can generate transpression and positive flower structures, a feature that we believe our seismic line has imaged for fault F_3_.

As the Centennial fault propagates westward, and the Lima Reservoir propagates eastward, they overlap forming an accommodation zone of opposing-dip oblique-slip normal faults. The inferred map pattern of these faults based on our subsurface data, matches the geometry of an oblique, anticlinal accommodation zone, described in Faulds and Varga^17^. The developing Centennial accommodation zone located directly west of our seismic line is similar in scale and function to others described in the CTB^41^ and elsewhere^11^. Specifically, growing fault tips of opposing dip are supported by map patterns and seismicity in the Red Rock Valley accommodation zone directly northwest of the Centennial Valley in southwestern Montana^13^. A 3-D block model developed for this structure (Fig. 7 in their paper)^13^ represents the model we have in mind for the Centennial Valley that is fully supported by the faults imaged in the seismic line (Figs 5–7).

As the Centennial accommodation zone developed, local transpression was realized on the curved fault segments. For example, antithetic fault F_3_ must have experienced down to south normal offset during the deposition of Unit 6 and the first part of Unit 7. During the latter part of unit 7 deposition, slip reversed, bringing the base of Units 3, 6 and 7 to near alignment, and the top of Unit 7 higher in the south than in the north. Such local transpressional offset is consistent with oblique slip along curved faults in the developing accommodation zone.

High-resolution seismic lines like the one we have acquired, processed and interpreted here, are crucial, if somewhat equivocal pieces of information in helping define the seismic hazards of a region. We note that with the exception of F_1_, none of the faults that we image in the seismic line ruptures the surface (Fig. 3D). This is also confirmed by the absence of evident high P-wave velocity bumps in our seismic tomography data (Figs 6, 7) that could pinpoint to presence of near-surface fault offsets^15^. Rather, all imaged faults seem to be sealed by Unit 9 and in some cases Unit 8, both of which we surmise to be late Pleistocene age. However, fault slip is continuing on blind faults because we see anticlinal bending in the zone surrounding F_3_, F_4_, and perhaps F_10_ (Figs 5 and 7). The lack of surface ruptures in the region directly surrounding our line is perplexing given the GPS geodetic velocities^3,5^, regional paleoseismic studies^6,7,12^ and rugged, youthful-appearing topography of the Centennial range front consistent with our ~0.2–0.6 mm/yr long-term fault slip. It is possible that the rate of deposition in the Centennial valley is rapid enough to outpace fault tip vertical propagation towards the surface. It is also possible that our line is situated near the centre of the accommodation zone where slip on individual fault segments is minimized. In summary, there are surface ruptures associated with the Lima Reservoir fault that align with the faults F_9_, F_10_, and F_11_ in Fig. 7 that lead us to conclude that we are witnessing lateral fault growth in assembling this accommodation zone.

The fact that GPS geodetic slip rates astride the Centennial Shear Zone are 10x faster than the paleoseismic slip rates for individual faults, like the Lima Reservoir fault, may not mean anything in terms of knowing which faults or fault segments that we have imaged with the seismic lines pose a significant seismic hazard. In a recent study Dolan and Meade^63^ compare the GPS geodetic interseismic slip to co-seismic slip for three large historic strike-slip earthquakes in comparable, sub-continental-scale shear zones. They find that in one case, the interseismic and co-seismic slip rates are comparable, in a second case the interseismic strain was much greater than the co-seismic slip, and in a third case, the co-seismic slip was much greater than the interseismic strain. Our seismic line illustrates that there are large basin bounding faults that are on projection with mapped fault segments (Fig. 2). Using well-known fault scaling relationships^64^ these faults are capable of Mo 6–7 earthquakes, which places the Centennial Valley into the same hazard potential as historic ruptures in the Hegben Lake (1959, Mw 7.3–7.5) and Lost River Valley earthquakes (1983, Mo 6.9). But the up-section diminished strata are offset on the largest faults, F1, F3, and F4, and the overall onlapping strata geometries of Units 8 and 9 seem to suggest slowing fault slip. One way to reconcile these observations is to suggest that earlier dip-slip motion on the Centennial and Lima Reservoir^65^ faults is being replaced by later strike-slip motion. The formation of the local inversion structure and accommodation zone is consistent with this interpretation and leaves open the possibility of co-seismic slip consistent with the interseismic GPS geodetic velocities.

## Conclusion

A new north-south oriented seismic line acquired across the Centennial Valley in southwestern Montana images a deformed basin stratigraphy consistent with basin development by both normal and strike-slip faults in the past 6 Ma. Fault geometries are consistent with a kinematic model of opposing fault tips on the north and south basin boundaries, propagating towards one another, leading to formation of an accommodation zone. The inferred geometry of the propagating faults based on our subsurface data, matches the geometry of an oblique, anticlinal accommodation zone and is also consistent with the transition from early dip-slip to more recent strike-slip motion on the faults. All of our results are supportive of GPS geodetic observations that identify the Centennial shear zone to be a major structure accommodating 1–3 mm/yr of right lateral shear across the NBR-SRP boundary.

## Supplementary information

Supplementary Material for main article

## Data Availability

The datasets analysed during the current study are available from the corresponding author on reasonable request.
