# Supplementary material for: Accommodation, slip inversion, and fault segmentation in a province-scale shear zone from high-resolution, densely spaced wide-aperture seismic profiling, Centennial Valley, MT, USA
Source: Sci Rep. 2019 Jun 25;9:9214. doi: 10.1038/s41598-019-45497-1 (PMC6593102; doi:10.1038/s41598-019-45497-1)
Supplement: Supplementary file 1 — Supplementary Material for main article [file 41598_2019_45497_MOESM1_ESM.docx]

Supplementary Material of

# Accommodation, slip inversion, and fault segmentation in a province-scale shear zone from high-resolution, densely spaced wide-aperture seismic profiling, Centennial Valley, MT, USA.

**Pier Paolo G. Bruno^1, *^, Claudio Berti^2,3^ and Frank J. Pazzaglia^2^**

^1^ Khalifa University, Department of Earth Sciences, P.O. Box 127788, Abu Dhabi, UAE;

^*^ pier.bruno@ku.ac.ae

^2^ Lehigh University, Department of Earth and Environmental Science, Bethlehem, PA 18015 USA

^3^ Idaho Geological Survey, Moscow, ID 83844 USA

Contents of this file

Text S1 to S4

Figures S1 to S4

Introduction

This document contains supporting information and figures for the main article. We summarize here the stratigraphy of the Centennial Valley and surrounding region; the field acquisition phase, the reflection processing flow used to produce the migrated stacks, and the inversion of the first break picks using the turning-ray tomography algorithm.

Text S1.

The figure below illustrates schematically the stratigraphy of the Centennial Valley and surrounding region. The Centennial Valley exposes stratigraphy of Precambrian to Quaternary deposits throughout the valley and adjacent mountain ranges. Precambrian basement rocks are exposed in the eastern Centennial Mountains and are overlain by a passive margin sequence of Paleozoic marine siliciclastic and carbonates from the middle Cambrian to the Permian^1^. The overlying Mesozoic sequence is a mix of marine and continental siliciclastics and carbonates deposited in the Sevier foreland^2^. These Mesozoic rocks are thickest in the western Centennial Mountains where the Frontier Formation is the main stratigraphic unit exposed^3^. North across the valley, basal conglomerates of the Beaverhead Group lie unconformably atop Paleozoic through mid-Mesozoic rocks^4^. Folds in the Beaverhead Group are evidence for syn-deformation deposition during the Sevier orogeny^2^. Unconformably overlying the Frontier Formation and Beaverhead Group are Cenozoic volcanic and volcaniclastic rocks of rhyolitic, andesitic, and basaltic composition of Challis and later Snake River Plain affinities. The younger part of this section is dominated by Yellowstone Pleistocene volcanic ash deposits which make up the south-dipping slope of the footwall of the Centennial Fault^5,1^.


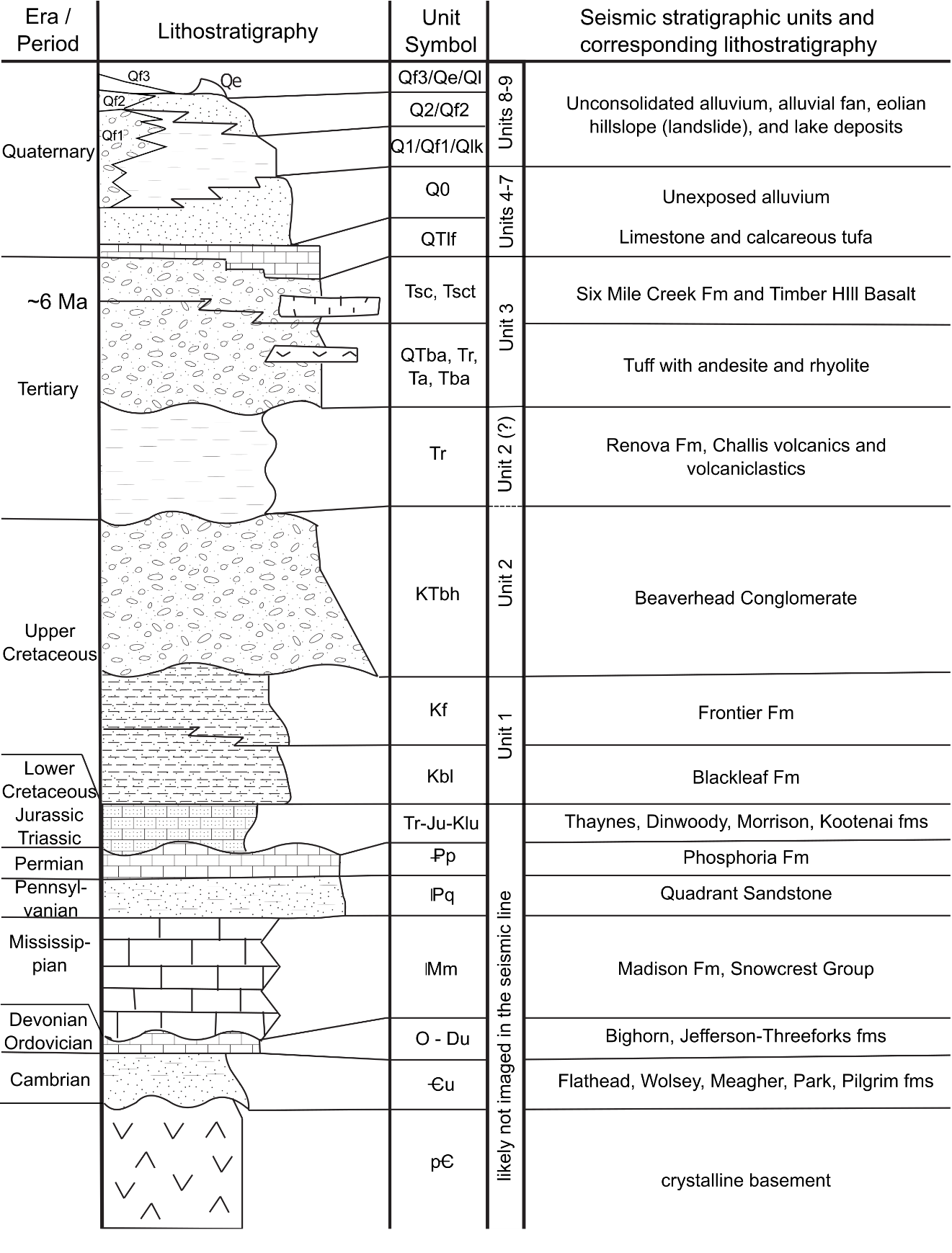


Figure S1. Generalized stratigraphic column of southwest Montana showing the age, symbol and name of each unit. Compiled from Lonn et al.^5^ and Hill et al.^6^ Detailed description of each unit found in Lonn et al.^5^

Text S2.

The figure below shows the overall pre-stack data quality and the acquisition parameters for the Price Creek and Matador lines, collected in the southern and northern sectors of the Centennial Valley, respectively. Four representative common-shot panels are in Fig. S2 A-D. They show high data quality, with high-frequency reflections (i.e. dominant frequency of ~80-120 Hz) and clear first arrivals in the entire offset range. Note also in the panels B and C a marked asymmetry of the basement reflection, compared to the overlying sedimentary reflections, suggesting a dipping basement morphology below the basin.


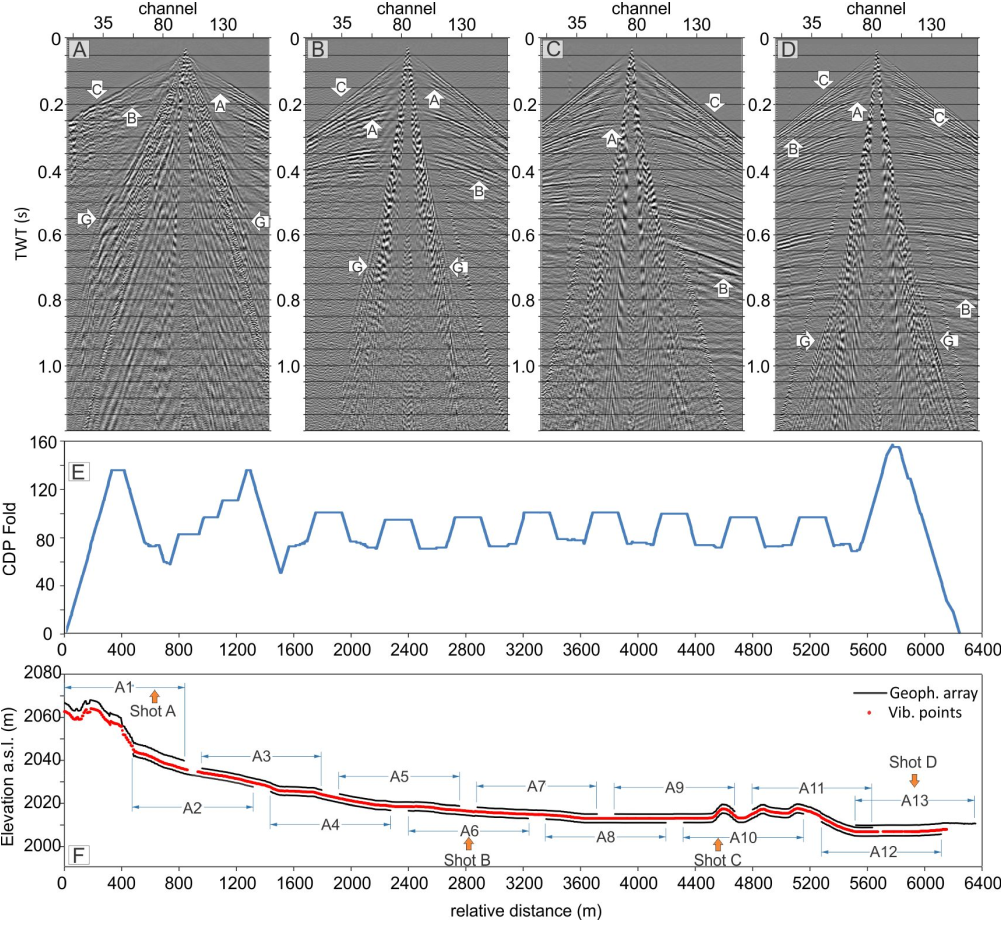


**Figure S2.** Upper panel (A,B.C & D): four common-shot gathers acquired in different locations along Price Creek line. The white arrows on the shot gathers outline: “A”; basement reflection beneath the basin “B”; high-angle refractions “C” and highly-dispersive surface waves, also known as “ground-roll “G”. Central Panel (E): CDP fold coverage along Price Creek profile. Maximum fold is 158 at CDP 5800; Bottom panel: (F) acquisition layout showing the thirteen wide-aperture geophone arrays used along Price Creek profile with all source locations (red dots).

To retain a high signal-to-noise ratio we planned to achieve a high data redundancy in the field (Fig. S2E). In order to maximize CDP fold, we fixed the 168-channel recording array length to be 835 m, and progressively moved the source at 5 m intervals within the fixed array, acquiring an average of 96 vibrating points. After the last shot in a sequence, the array was shifted towards the north (to the right in panel F) by 96 geophone positions, leaving an overlap area of 72 geophones between two adjacent arrays. This overlap was necessary to minimize the loss of data redundancy at changeovers. The procedure was repeated for 13 arrays for the Price Creek and 5 arrays for the Matador lines. The resulting CDP fold is very high, with average values of ~82 traces per CDP and maximum values exceeding 150 traces. CDP fold decreases evenly at both ends of the profile.

The last panel (Fig. S2F) shows the elevation pattern and source-receiver positions along Price Creek profile. Both profiles were acquired along a S-N direction. The Price Creek profile was acquired using 1264 geophone positions each spaced 5 m apart. The change in elevation is 56 m, with a maximum altitude of 2063 m at the southern end of the line and a minimum elevation of 2007 m where the profile crosses the Red Rock River, near the northern end of the line. The Matador line was acquired using 640 geophone positions. Elevation variations along this profile are minimal (i.e. less than 25 m over 3056 m distance).

Text S3.

The figure below illustrates schematically the data processing flow used in processing the seismic data. The upper central side of the diagram (blue) illustrates the processing steps applied to correlate the raw field data and prepare them for first arrival picking, which is needed for refraction static correction. The lower central side of the diagram (green) illustrates the algorithms applied for reflection processing (pink). The right side of the diagram show instead the stacking velocity model building and stacking process, consisting of semblance, residual statics DMO and stacking. Final CMP stack has been migrated using a post-stack Kirchhoff algorithm. A smooth and consistent velocity model along the entire seismic profile length is required to develop a reliable depth migrated image^7^. In layer-based models, velocity gradients are bounded by sedimentary interfaces. There, it is sufficient to pick seismic-reflection event. In our case, continuous coherent reflections fill the entire basin. In the southern side of our profile, especially in the Centennial Fault footwall, the reflected arrivals are shallower and are recorded over the ground roll window in a contest in which the velocity field does not follow continuous reflections. In this part of the profile, we needed an “a priori” assumption of how the velocity field behaves. This information has been provided by our refraction tomography results (see text and Fig. S43).


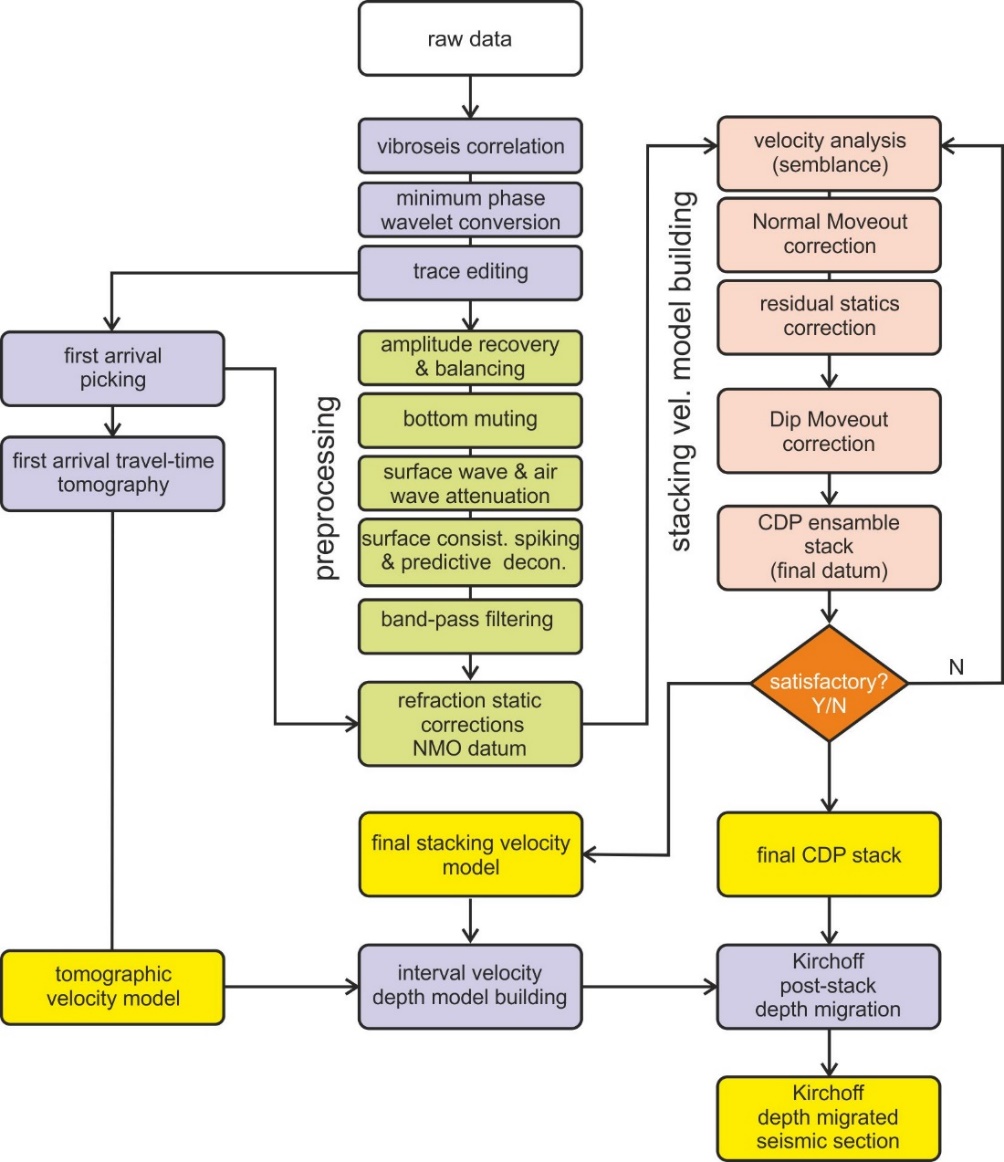


**Figure S3**. Data processing flow.

**Table S3:** Seismic data processing streams used


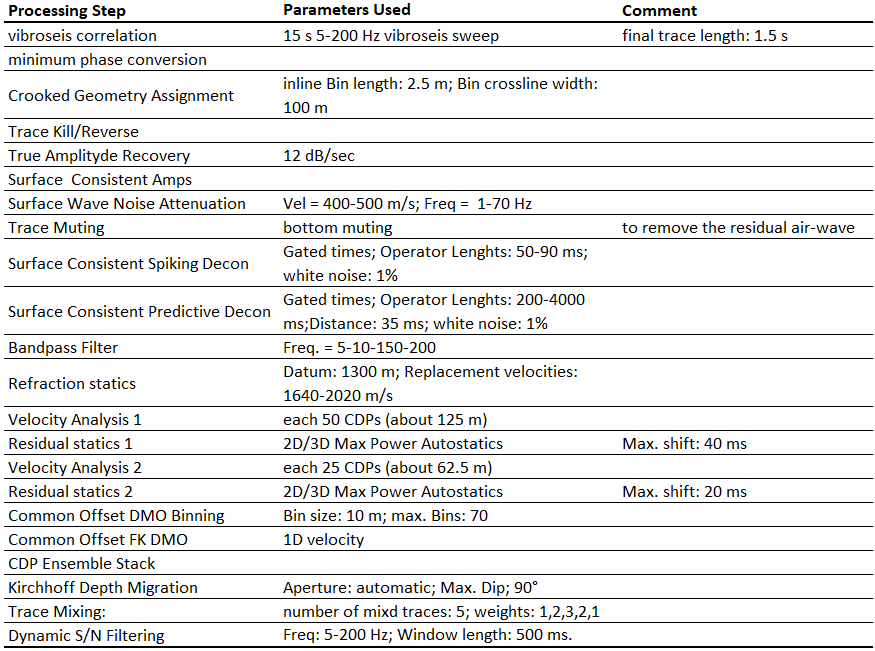


Text S4.

The use of dense, wide-aperture arrays allowed us to record highly redundant, global-offset first arrivals that were further checked for consistency using the reciprocity rules of Ackermann et al.^8^ For each of the two profiles, we built an accurate near surface velocity model through the inversion of the first break picks using the turning-ray tomography technique^9^. This tomographic algorithm solves the direct problem by tracing turning rays through an “a priori” model, which in our case was estimated using a time-delay refraction method.

The velocity models were discretized on a 10x10 m grid size, dependent on the field recording parameters (i.e. geophone spacing and source move-up) and it is chosen in order to obtain the best compromise between the model resolution and ray density (number of ray segments within each cell). In our case the high-density of sources and geophones allowed us to achieve a very large ray density and a very detailed and reliable P-wave velocity field. The theoretical ray paths and the travel-time residuals were then iteratively inverted using a SIRT-based method^10^ to produce the two-dimensional velocity field that best minimizes the travel-time residuals. The ray density 2D distribution (Fig S3, bottom), together with the RMS value of travel-time residuals were used as criteria to evaluate stability and robustness of the solutions. For Matador line the inversion stopped after 24 iterations; the minimum RMS error was 2.66 ms. For Price Creek line a minimum RMS error 3.06 ms was reached after 30 iterations over a total of 32 iterations.

**
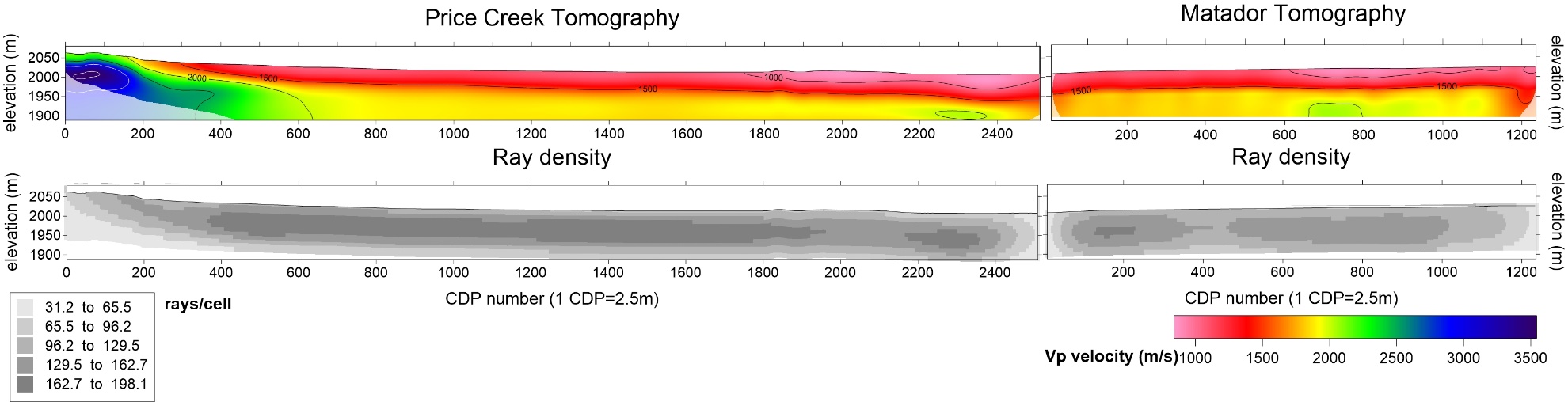
**

**Figure S4**. Top figures: results of Vp refraction tomography along Price Creek (left) and Matador (right) lines. Bottom figures: distribution of number of rays per cell. Vertical and horizontal scales are equal.

**References**.

- - 1. Witkind, I. J. Geologic map of the Centennial Mountains wilderness study area and contiguous areas, Montana and Idaho. *U. S. Geological Survey Miscellaneous Field Map* MF-1342-A1. Scale 1;50,000 (1982).
    2. Majerowicz, C.N., Troy, J. K., Anastasio, D. J., and Pazzaglia, F. J. Bedrock and surficial geologic map of the Lima Dam 7.5’ quadrangle Beaverhead County, Southwestern Montana.  *Montana Bureau of Mines and Geology* *EDMAP.* **7**, 17 p., 2 sheets, scale 1:24,000 (2010).
    3. Petrik, F. E. Scarp analysis of the Centennial normal fault, Beaverhead County, Montana and Fremont County, Idaho. *M.S. Thesis, Montana State University, Bozeman, MT*. 287 p. (2008).
    4. Dyman T.S., R.G. Tysdal, W.J. Perry Jr., D.J. Nichols, J.D. Obradovich. Stratigraphy and structural setting of Upper Cretaceous Frontier Formation, western Centennial Mountains, southwestern Montana and southeastern Idaho. *Cretaceous Research.* **29**, 237-248 (2008).
    5. Lonn, J.D. et al. Geologic map of the Lima 30' x 60' quadrangle, southwest Montana, Montana. *Bureau of Mines and Geology: Open-File Report.* **408**, 12 p., 1 sheet(s), 1:100,000 (2000).
    6. Hill, C.L. The Merrell Locality (24BE1659) & Centennial Valley, Southwest Montana: *Pleistocene Geology, Paleontology & Prehistoric Archaeology. Billings, MT*: BLM Montana State Office (2005).
    7. Yilmaz, O. Seismic Data Analysis (2 Volumes), Society of Exploration Geophysicists. *SEG Investigations in Geophysics.* **10**, 1000 p. (2001).
    8. Ackermann, H. D., Pankratz, L. W., and Dansereau, D. Resolution of ambiguities of seismic refraction traveltime curves. *Geophysics*. **51**(2), 223-235 (1986).
    9. Stefani, J.P. Turning-ray tomography. *Geophysics.* **60**, 1917-1929 (1995).
    10. Epili, D., J. Criss, D. Cunningham. Turning-Ray Tomography for Statics Solution, *Society of Exploration Geophysicists Annual Meetin*g, 9-14 (2001).
